# Prevalence of tuberculosis, multidrug resistant tuberculosis and associated risk factors among smear negative presumptive pulmonary tuberculosis patients in Addis Ababa, Ethiopia

**DOI:** 10.1186/s12879-019-4241-7

**Published:** 2019-07-19

**Authors:** Waganeh Sinshaw, Abebaw Kebede, Adane Bitew, Ephrem Tesfaye, Mengistu Tadesse, Zemedu Mehamed, Bazezew Yenew, Misikir Amare, Biniyam Dagne, Getu Diriba, Ayinalem Alemu, Muluwork Getahun, Dinka Fikadu, Kassu Desta, Habteyes Hailu Tola

**Affiliations:** 1 Ethiopian Public Health Institute, Addis Ababa, Ethiopia; 2 Department of Medical Laboratory Science, College of Health Sciences, Addis Ababa University, Addis Ababa, Ethiopia

**Keywords:** Prevalence, Smear negative PTB, Smear negative MDR TB, Xpert MTB/RIF assay

## Abstract

**Background:**

The diagnoses of active smear negative PTB, remains difficult. As a result, treatment is often carried out empirically relaying on clinical criteria. The distribution and magnitude of smear negative PTB, smear negative MDR-TB and associated factors in the same day diagnosis strategy are not clearly known in the study area. Therefore, this study aimed to determine the prevalence of TB, MDR-TB and associated risk factors among presumptive smear negative pulmonary tuberculosis patients in Addis Ababa, Ethiopia.

**Methods:**

Analytic cross sectional study design was used. A total of 418 smear negative presumptive pulmonary TB patients were enrolled from selected health facilities since August 01, 2017 to January 5, 2018. Sputum samples were examined by Ziehl Neelsen microscopy, Xpert MTB/RIF assay and Culture. Drug susceptibility testing was performed by line probe assay and BACTEC MGIT 960 system. These laboratory tests were performed in Ethiopian Public Health Institute, National TB Reference Laboratory. Data was analyzed by SPSS Ver.20.

**Results:**

From the total of 418 enrolled patients, 27 (6.5%) were Xpert MTB/ RIF and 26 (6.4%) were culture confirmed smear negative PTB patients. The positivity rate among male and female was 10.2 and 3.5% (*p* = 0.005) respectively. From 26 culture positive isolates 3 (11.54%) were MDR TB; from MDR-TB confirmed isolates 2/23 (8.7%) were among new and 1/3 (33.3%) was among retreatment smear negative presumptive pulmonary TB patients. All Rifampicin resistant smear negative pulmonary TB isolates by Xpert MTB/ RIF assay were found to be MDR TB and 7/26 (26.9%) isolates were INH mono resistant. History of migration found to be a potential factor for developing smear negative pulmonary TB.

**Conclusion:**

In this study a significant proportion of smear negative pulmonary TB was diagnosed. Furthermore, a high smear negative multi drug resistant (MDR) TB and other mono drug resistant TB prevalence was confirmed. Due to the limitations of smear microscopy which is used as a primary diagnostic tool, these TB strains are missed to be diagnosed and transmission continues in the community.

## Background

Tuberculosis (TB) continues as a challenging bacterial disease. In 2017 alone, 10 million people have developed TB disease [1]. TB is the leading cause of death in the world among infectious diseases [1]; 1.3 million died among HIV negative and 300, 000 among HIV positive people due to TB by the year 2017 [1]. One person dies every 15 s in the world by TB and it infects one person every second of every day. If treatment is not initiated, a person with TB disease will infect an average of 10–15 other people every year [2]. In addition, emergency of drug-resistant TB makes the global TB control program more difficult. An estimated 3.5 and 18% are multidrug-resistant (MDR)/rifampicin resistant (RR) TB among new and previously treated TB patients respectively [1]. Moreover, about 9% of MDR-TB patients also have extensively drug-resistant TB (XDR-TB) [3]. Ninety-five percent of all TB diseases and 99% of deaths occur in developing countries, with the greatest burden in sub Saharan Africa [4]. Ethiopia is one of the 30 high TB; TB/HIV and MDR TB burden countries with an estimated TB incidence of 164 per 100,000 population of drug susceptible TB [1]. In addition, the burden of MDR-TB in Ethiopia is increasing. In 2005 the national prevalence of MDR-TB was 1.6% among new patients and 11.8% among previously treated patients [5]. However, in 2014 the national prevalence of MDR-TB was 2.3% among new and 17.8% among previously treated patients [6].

WHO recommends smear microscopy as an initial TB diagnostic tool. However, it cannot detect 43% of TB patients due to its poor sensitivity [7]. Its sensitivity is further reduced in patients with extra pulmonary TB, children and in those who are co infected with HIV*.* Furthermore, it cannot distinguish drug-susceptible from drug-resistant strains [8]. TB culture confirmation has a long turnaround time and needs high safety requirement [8]. On the other hand, molecular methods show variable sensitivity [8]. Due to the above reasons TB diagnosis faces a huge challenge*.* In particular, the diagnosis of smear negative pulmonary TB (SNPT) and smear negative drug resistant TB (SNDRT) is the most challenging in under developed countries [9]. Therefore, as a result this treatment is often given empirically using clinical criteria which leads to miss diagnosis of the patients, unnecessary costs, toxicities, delayed response and poor treatment outcome [10].

In 2017, 3.6 million TB patients left undiagnosed and only 25% of the estimated MDR/RR-TB was reported [1]. Smear negative pulmonary TB (SNPT) is a critical clinical and public health problem [11, 12]. This SNPT prevalence can reach 36 to 45% in United States of America [13], 65.2% in Brazil among high HIV prevalence setting (41.1%) [9], 68.1% in Italy [14], 55% in Belarus (from this 47% were smear negative MDR TB) [15]. Studies in Ethiopia have shown different evidences of SNPT prevalence. A study conducted in Adama, in 2013, indicated that from 232 sputum smear negative presumptive pulmonary tuberculosis, 28 (12.1%) prevalence of smear negative pulmonary tuberculosis [16]. Similarly, a study in North Shoa, Ethiopia has also shown a high prevalence (23.9%) smear negative pulmonary TB [17]. MDR TB among smear negative presumptive pulmonary TB patients is not reported yet in Addis Ababa. A study conducted in St. Peter TB specialized hospital Addis Ababa between 2004 and 2005 didn’t find MDR TB from smear negative TB isolates [18]. The mortality rate of SNPT can reach 25% in populations with high prevalence of HIV infection, which may be largely related to delay in diagnosis [12, 19, 20]. Furthermore, 10–20% of TB transmission at the population level is attributable from SNPT patients [21, 22]. Patients with smear-negative pulmonary TB were found to be less infectious and to have a lower mortality but a significant proportion (50–71%) progressed to active TB disease [21, 23].

Different risk factors have different impacts on the occurrence and diagnosis of SNPT. Studies have shown different risk factors for the occurrence of SNPT [9, 24, 25]. But, there is limited information about factors associated with SNPT in the study area.

There is a limited data on the prevalence of smear negative pulmonary tuberculosis, smear negative multidrug resistance tuberculosis and associated risk factors among smear negative presumptive pulmonary tuberculosis patients in Addis Ababa, Ethiopia. Therefore, the aim of this study was to determine the prevalence of tuberculosis, multidrug resistance tuberculosis and associated risk factors among smear negative presumptive pulmonary tuberculosis patients in the above study area.

## Methods

### Study design, study site and population

**Fig. 1:**
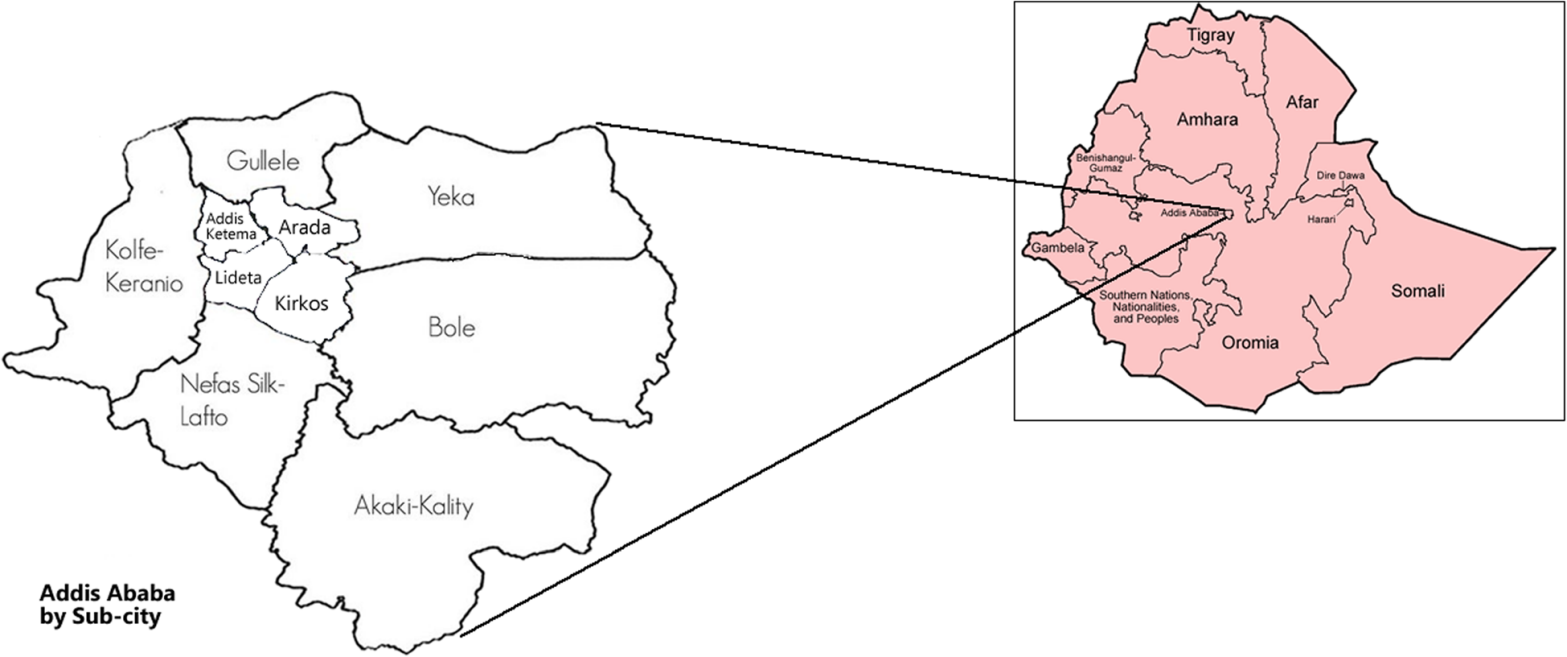
Map of Addis Ababa

This is an analytic cross sectional study which was conducted in Addis Ababa, Ethiopia from August 2017 to January, 2018. Addis Ababa is the Federal Capital of Ethiopia. It is located between 8055′ and 9005′ North Latitude and between 38040′ and 38050′ East Longitude. The population is more than 3 million [26] (Fig. 1).

The study was conducted in 16 governmental and private health facilities included in directly observed treatment short course (DOTS) program in Addis Ababa region by the year 2017. A list of all DOTS sites was prepared on excel sheet and rand command was used to select health facilities from the list. Then by using simple random sampling technique 20% of the sites were selected out of the lists. Simple random sampling was used to select study sites since there were similar characteristics among governmental and private DOTS sites.

Sputum smear negative presumptive pulmonary tuberculosis patients who were equal or greater than the age of 2 (two) years visiting the selected health facilities during the study period were included in the study. Whereas, those who were taking anti-TB drugs for greater than 1 week by any means and patients incapable of producing sputum were excluded.

### Sample size determination

Sample size calculation was performed for both objectives; prevalence of smear negative pulmonary TB and MDR TB by using single population proportion formula with corrected. Then the larger sample size was taken to represent the other. For prevalence of smear negative pulmonary tuberculosis, the sample size was 264 and for prevalence of SNMDR TB, it was 413 in 95% confidence interval. Therefore the 413 was the total sample size for this study [6].

### Data collection procedure

Onsite training was given for all laboratory staffs and clinicians about the project for each respective study sites. Data was collected using structured questioner about socio-demographic information, clinical presentations (Table 5) and comorbidities/co-infections (HIV AIDS, diabetes mellitus, asthma, hypertension, heart disease, liver disease, renal failure, hormonal disease, malignancy/ cancer), behavioral and other risk factors (miners, prisoners/prisoner staffs, migrant, TB/MDR TB contact, alcohol consumption, smokers, and chewing Khat) from smear negative presumptive pulmonary tuberculosis (SNPPT) patients. Laboratory investigation data was also taken from study registration book, printouts, and culture work sheet.

Two spot sputum specimens (in Spot-Spot sample collection strategy) were collected from, presumptive pulmonary tuberculosis patients and checked for AFB using ZN method in the health facilities. Specimen was collected for the purpose of the study from smear negative presumptive pulmonary patients and from left over samples which were collected from presumptive pulmonary TB patients as well. All patients who were smear negative were enrolled according to the inclusion criteria. Sputum sample was collected with wide mouthed, clean, 50 ml capacity, sterile, disposable containers, polypropylene centrifuge tubes, translucent, screw capped container after detail patient instruction at the health facility level. It was expected to collect sample with volume > = 3 ml; but those samples less than 3 ml was rejected, wrong sample containers, containers with leakage, breakage, and with incomplete questioner were rejected. Specimens collected was stored at 2-8^o^c until transported. The specimen was transported using triple packaging. Specimens arrived at the Ethiopian Public Health Institute (EPHI), National Tuberculosis Reference Laboratory (NTRL), was rechecked for fulfilling criteria and was stored again at 2-8^o^c in the refrigerator if it was not processed immediately.

### Laboratory investigations

**Fig. 2:**
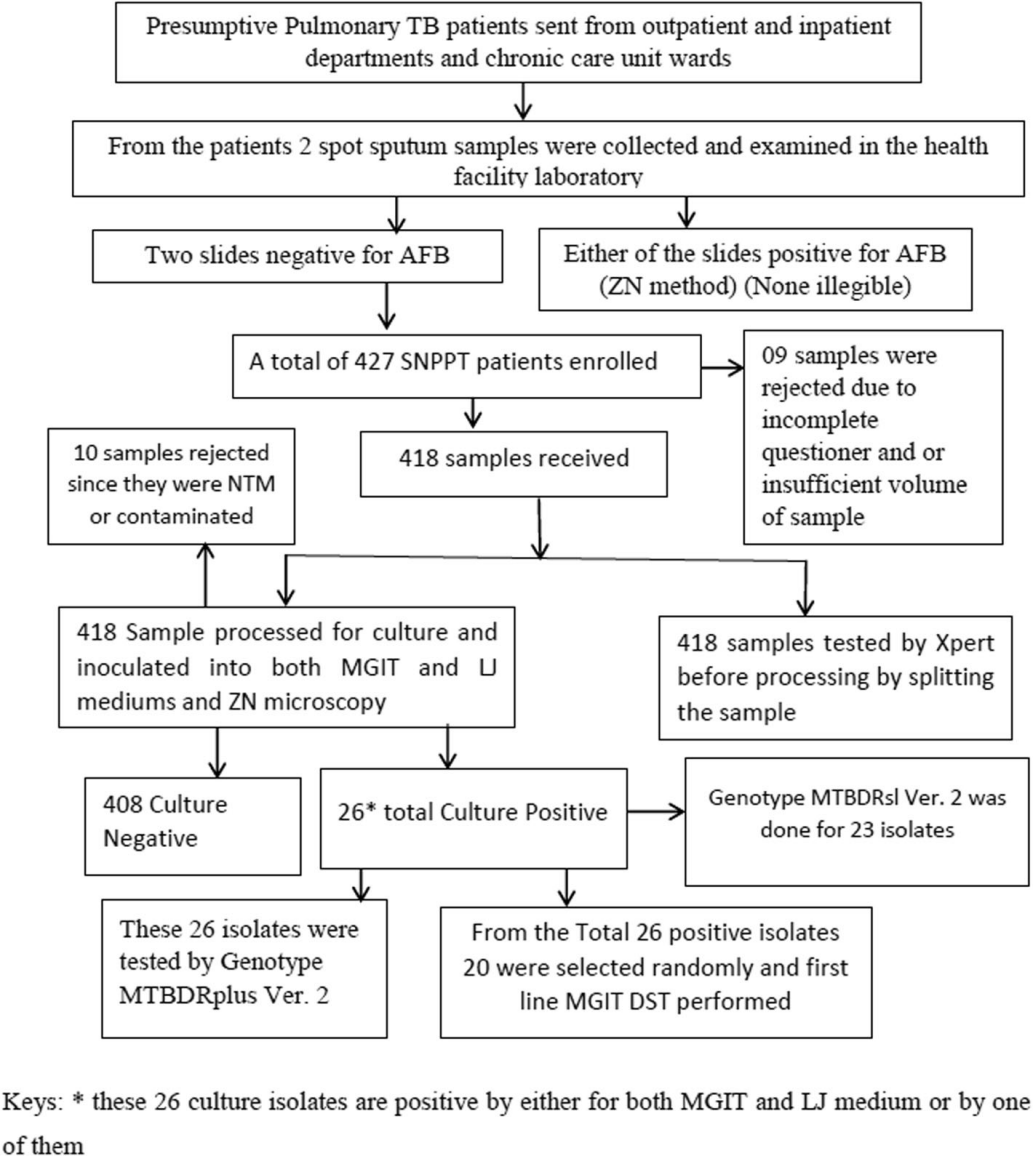
Flow chart of the study

From the specimen collected, AFB microscopy [27], Xpert MTB/RIF assay (Cepheid, Sunnyvale, CA, USA) (directly from the sample before processing for culture), Liquid (BACTEC MGIT 960 (Becton-Dickinson and Company, Sparks MD)) and Solid culture (LJ media) were performed in the EPHI, NTRL. Drug susceptibility testing was performed here for the culture isolates. Any result found positive was reported to the physician requested the test for further patient management. The samples were splited in to two; one for culture and the other for Xpert MTB/ RIF assay. For Xpert MTB/ RIF assay, sample was mixed with sample reagent buffer in 1:2 (sample: sample reagent buffer) volume ratio. Then, closing it tightly, vortexed for 15 s and allowed to stand at room temperature for 10 min. It was vortexed again after 10 min and allowed to stand for 5 min, totally for 15 min. Using the Pasteur pipette provided with the kit > 2 ml of the (just above 2 ml mark on pipette) processed sample was put into the Xpert MTB/RIF cartridge. After that, the cartridge with the specimen was loaded to the GeneXpert machine. Finally, results were collected from the GeneXpert computer after 2 h [28]. Samples for culture were processed with 3% N-Acetyl-L-Cysteine–Sodium Hydroxide (NALC-NaOH) decontamination method. Equal volume of NALC-NaOH-sodium citrate solution was added to the sample, vortexed and kept stand at room temperature for 15 min to let the NaOH decontaminate normal flora present in the sputum with the help of the mucolytic agent. Then, this solution was neutralized (effect of NaOH stopped) by 6.8 pH phosphate buffer solution (PBS), concentrated the freed bacilli in the tube by centrifugation at relative centrifugal force of 3,000 x g for 15 min using safety centrifuge at a temperature of 4–8 °C. After this the supernatant removed, smear made from the sediment and sediment resuspended by adding 2 ml of PBS. Making the sediment uniform by vortexing, 0.5 ml and 2–4 drops were added to the MGIT tube and LJ tube respectively. The MGIT tube was enriched with 0.8 ml of a mixture of growth supplement which contains Middle brook OADC enrichment and PANTA (lyophilized mixture of antimicrobial agents) prior to the inoculation of the tube with the sediment. After all, the LJ medium incubated in the incubator at 37 °C and the MGIT tube loaded to the BACTEC MGIT 960 machine. For positive culture results smear prepared and blood agar/ brain heart infusion agar inoculated for positive MGIT tube and smear prepared and examined for positive LJ for identification mycobacterium TB complex (MTBC) from other non-tuberculous mycobacteria and contaminants. Negative results reported after 42 days for MGIT 960 system and 56 days for LJ medium [29]. SD BIOLINE TB Ag MPT 64® (STANDARD DIAGNOSTICS, INC, Republic of Korea) test was used for the confirmation of MTBC [30]. Genotypic and phenotypic drug susceptibility testing was performed using world health organization recommended methods: line probe assay (MTBDR *plus* Ver.2 for first line anti-TB drugs, MTBDR*sl* Ver.2 for second line anti TB drugs) and BACTEC MGIT 960 system (for first line drugs using proportion method) respectively (Fig. 2).

### Quality assurance and quality control

Reagents, and culture media prepared within the laboratory or purchased as readymade which have effect on the test result was checked for sterility and performance characteristics using well characterized control slide or strains such as; a positive and negative control smear slides were included in each batch of new lots of reagents prepared, 2% of a batch of tubes of MGIT Culture media was tested with different dilutions of M. tuberculosis (H37Rv). Specimens which had breakage or leakage, incomplete patient information and mislabeling, delayed specimen more than 5 days after collection, inappropriate container, and insufficient volume of sample (less than 3 ml) were rejected. Processing reagents (NaOH, Phosphate buffer) was sterilized before use. Each batch of samples that was processed for culture used start and end controls. All laboratory investigations were done by experienced laboratory technologists in the National Tuberculosis Reference Laboratory, EPHI. Each of the culture and identification procedures was performed in a certified class II Bio-Safety Cabinet. Equipment preventive maintenance, temperature monitoring, monitoring of critical microbiological practices, and instrument function checkups was done. The laboratory performance is also continuously monitored by Proficiency Testing (PT) for all test methods. All laboratory analysis was done based on Ethiopian national TB, HIV and leprosy management guideline and WHO approved methods.

### Data entry and analysis methods

The data collected from each questionnaire and the laboratory investigation was doubly entered and cleaned by using Epi -data statistical software version 3.0 and exported to IBM SPSS software version 20.0 for analysis. By using central measurement, dispersion and frequency distribution descriptive statistics was done to characterize the study participants and the result of the study was also presented by using tables and figures. The prevalence of SNPT and SNMDR TB was calculated with its 95% CI. Binary logistic regression also done to determine the association between dependent variables and possible risk factors. Variables which show significance at *P*-value of 0.2 during univariate analysis were selected for multivariable analysis. The strength of association was measured by using odds ratio and P*-*value of less than 0.05 was considered as statistically significant during multivariable analysis.

## Results

### Socio demographic characteristics of participants

**Fig. 3:**
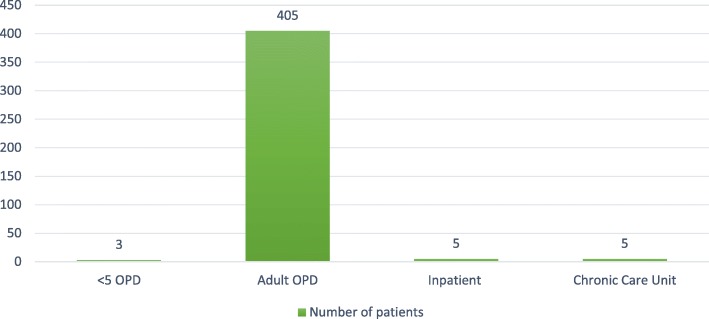
A bar graph showing study participant proportion by department

A total of 418 patients’ epidemiological and bacteriological data were analyzed. Most of the patients were from adult out-patient-department (OPD) (Fig. 3). Majority, 231(55.3%) of the patients were females. The mean age of participants was 35.68 (± 17.12) years. Most of the participants, 125 (29.8%) were in the age group of 25–34 years, and children under 15 years were 20 (4.8%). The predominant religion was Orthodox Christians, 323 (77.3%), followed by Muslims, 57 (13.6%). Among all participants, 228 (nearly 55%) were below grade 7. The participants had different occupation; the most frequent was house wife, 87 (21.1%) and the second frequent work was daily laborer, 76 (18.4%). Two hundred eighteen (52.3%) participants were married whereas 159 (38.1%) were single (Table 1).

**Table 1:** Socio demographic characteristics of study participants in Addis Ababa, August 01, 2017- January 05, 2018

Variables	Frequency	Percent (%)
Sex		
Male	187	44.7
Female	231	55.3
Total	418	100
Age Group		
0–14	20	4.8
15–24	94	22.4
25–34	125	29.8
35–44	61	14.6
45–54	53	12.6
55–64	27	6.4
> 65	38	9.3
Total	418	100
Religion		
Orthodox	323	77.3
Muslim	57	13.6
Protestant	34	8.1
Catholic	2	0.5
Other	2	0.5
Total	418	100
Level of Education		
None(No Formal Education)	112	26.9
Standard grade 1–7	116	27.9
Standard grade 8–10	88	21.2
Standard grade 11–12	53	12.7
Diploma(level 1–4 Certificate)	26	6.3
First Degree	21	5.0
Second Degree & above	0.0	0.0
Total	416	100
Work status		
Farmer	31	7.5
Daily Laborer	76	18.4
House wife	87	21.1
Driver	10	2.4
Teacher	7	1.7
Student	46	11.2
Merchant	14	3.4
Health Care Worker	3	0.7
Government employee	35	8.5
Self-employed	60	14.6
Other	43	10.4
Total	412	100
Marital Status		
Single	159	38.1
Married	218	52.3
Divorced	22	5.3
Widowed	16	3.8
Separated	2	0.5
Total	417	100

### Clinical presentation, comorbidities and associated risk factors

**Fig. 4:**
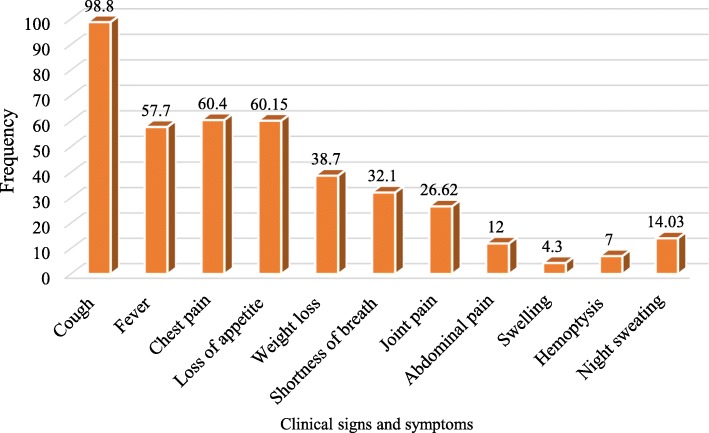
Clinical presentations among study participants

**Fig. 5:**
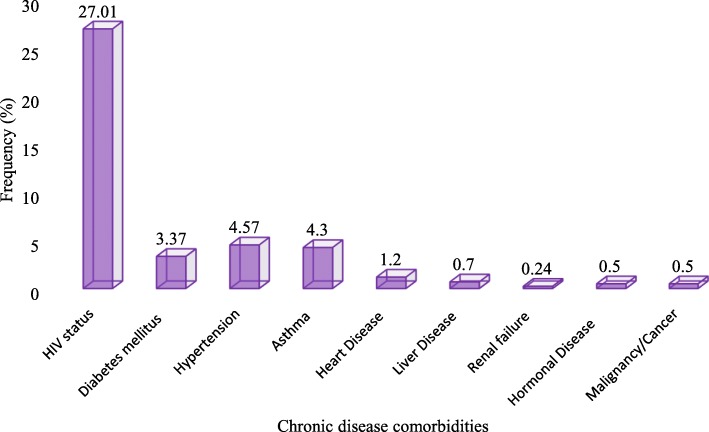
A bar graph demonstrating frequency of chronic comorbidities among study participants

Signs and symptoms, comorbidity (self-reported by the patient not confirmed) and other risk factors were assessed in the study. From all the participants, 367 (88%) had cough as a chief compliant, and 112 (30.5%) of them with productive cough. HIV status was known only for 211 patients. HIV positivity rate was 27%. Fourteen (3.37%) participants were known to have diabetes mellitus (DM). Thirty five (8.4%) patients had TB contact history (Figs. 4 and 5, Table 2).

**Table 2:** Behavioral and other risk factors, Addis Ababa, Ethiopia, August 01, 2017- January 05, 2018

Risk factors	Frequency	Percent (%)
Miner	11	2.7
Prisoner/Prisoner staff	2	0.5
Migrant	11	2.7
TB/MDR TB contact	35	8.45
Alcohol Consumption	122	29.3
Smokers	26	6.3
Chewing Khat	57	13.7

### Prevalence of smear negative pulmonary tuberculosis (SNPT)

SNPT prevalence among presumed smear negative PTB patients was 6.5% (95% C.I, 4.103–8.816) by Xpert MTB/RIF assay. Twenty six patients (6.4%) were smear negative and culture confirmed / by either of the culture techniques (LJ and MGIT), SNPT patients (Table 3).

**Table 3:** Detection of TB using different diagnostic tools among presumptive smear negative pulmonary TB patients, August 01, 2017- January 05, 2018

Diagnostic Method	Negative Result No. (%)	Positive Result No. (%)	Total No. (%)	Contaminated (%)	NTM (%)
Xpert MTB/RIF assay	391 (93.5)	27 (6.5)	418 (100)	NA	NA
MGIT culture result	354 (92.65)	24 (6.35)	378 (100)	34 (8.25)	06
LJ culture result	376 (94.47)	22 (5.53)	398 (100)	18 (4.3)	01
LJ and MGIT culture result	382 (93.6)	26 (6.4)	408 (100)	03 (0.73)	07

More TB was detected in male than female patients. The positivity rate among male and female was 10.2 and 3.5%, respectively. There was significant difference in TB prevalence among male and female (*p* = 0.005). High rate of TB detection was observed in the age range 15–24 years, 8 (8.5%) and 25–34 years, 11 (8.9%). All positive SNPT were detected from the age groups between 15 and 64 years. SNPT prevalence was, 20 (6.2%) among Orthodox Christians, 5 (8.8%) and 2 (6.0%) among Muslims and Protestants respectively. Majority, 21 (80.77%) of the total SNPT were found to be positive in those who were below grade 12, but it was not statistically significant (*P* = 0.496). From the total of 411 participants responded, the most common work status, 87 (21.1%) was house wife; however SNPT prevalence was low, 01 (1.2%). The second most frequent work, 76 (18.4%) was daily laborer with (7.9%) SNPT prevalence, and the least frequent, 03 (0.7%) was health worker, with the highest SNPT prevalence 01(33.3%) and there was an association with the SNPT (*P* <  0.006). The odds of having SNPT was 68.7 times higher in health care workers as compared to farmers, (AOR 68.7; 95% C.I, 2.447–1929.15, *p* = 0.013). From 417 participants interviewed, 218 (52.3%) were married, from which 11 (5.04%) was TB positive, 159 (38.1%) were single from which 14 (8.8%) was TB positive, (*p* = 0.460) (Table 4).

**Table 4:** Socio demographic characteristics of study participants and level of Association (Chi square), August 01, 2017- January 05, 2018

Variables	Positive (*n* = 27)	Negative (*n* = 391)	*P*-value
Sex			
Male	19	167	0.005
Female	8	223	
Total	27	390	
Age Group			
0–14	0.0	20	0.237
15–24	8	86	
25–34	11	113	
35–44	05	56	
45–54	01	52	
55–64	02	25	
> 65	0.0	38	
Total	27	390	
Religion			
Orthodox	20	303	0.935
Muslim	5	52	
Protestant	2	31	
Catholic	0.0	02	
Other	0.0	02	
Total	27	390	
Level of Education			
None(No Formal Education)	06	106	0.496
Standard grade 1–7	04	112	
Standard grade 8–10	06	82	
Standard grade 11–12	05	48	
Diploma(level 1–4 Certificate)	03	23	
First Degree	02	18	
Second Degree & above	0.0	0.0	
Total	26	389	
Work status			
Farmer	01	30	0.006
Daily Laborer	06	70	
House wife	01	86	
Driver	0.0	10	
Teacher	0.0	07	
Student	0.0	46	
Merchant	0.0	14	
Health Care Worker	01	02	
Government employee	02	32	
Self-employed	07	53	
Other	07	36	
Total	25	386	
Marital Status			
Single	14	145	0.460
Married	11	206	
Divorced	02	20	
Widowed	0.0	16	
Separated	0.0	02	
Total	27	389	

This was done based on both GeneXpert and culture results

Of 417 participants, 345 (82.73%) were new presumptive TB patients and 23 (6.67%) had confirmed TB. On the other hand, 72 (17.27%) participants were retreatment presumptive TB patients and 4 (5.56%) of them were positive for TB. There was significant difference in TB detection among new and retreatment presumptive TB patients (*p* <  0.001).

Among clinical signs and symptoms this study investigated; weight loss (*p* < 0.001), shortness of breath (*p* = 0.034) and loss of appetite (*p* = 0.023), having association with the SNPT in univariate analysis. However, in the multivariate analysis these symptoms are not statistical significant; loss of appetite (AOR 0.778; 95% C.I, 0.248–2.438, *P* = 0.666), weight loss (AOR 0.340; 95% C.I, 0.108–1.069, *p* = 0.065) and shortness of breath (AOR 0.493; 95% C.I, (0.181–0.1.343), *p* = 0.167). Known chronic malignancies **(**Table 5**)** and comorbidities were not significantly associated with SNPT depending on these study findings. HIV-TB co-infection was 5.3%. From behavioral and other risk factors that showed association in the univariate model, only being migrant were statistically significant factor for the SNPT (AOR 0.121; 95% C.I, (0.20–0.730) *p* = 0.021) (Table 6).

**Table 5:** Clinical presentation, comorbidities and associated risk factors for SNPT & SNMDR TB, August 01, 2017- January 05, 2018

Variables	Yes/No	Negative frequency (%)	Positive frequency (%)	*P*-value
Signs and symptoms				
Cough	Yes	385 (93.4)	27 (6.6)	0.554
	No	05 (6.4)	0 (0.0)	
Fever	Yes	236 (92.5)	19 (7.5)	0.317
	No	153 (95%)	8 (5.0)	
Chest pain	Yes	232 (92.1)	20 (7.9)	0.134
	No	158 (95.8%)	07 (4.2)	
Loss of appetite	Yes	222 (91.4)	21 (8.6)	0.034
	No	168 (96.6%)	06 (3.4)	
Weight loss	Yes	141 (87.6)	20 (12.4)	< 0.001
	No	248 (97.3%)	07 (2.7)	
Shortness of breath	Yes	120 (89.6)	14 (10.4)	0.023
	No	270 (95.4%)	13 (4.6)	
Joint pain	Yes	102 (91.9)	09 (8.1)	0.414
	No	288 (94.1)	18 (5.9)	
Abdominal pain	Yes	48 (96.0)	02 (4)	0.448
	No	342 (93.2)	25 (6.8)	
Swelling	Yes	17 (94.4)	01 (5.6)	0.871
	No	373 (93.5)	26 (6.5)	
Hemoptysis	Yes	25 (86.2)	04 (13.8)	0.098
	No	364 (94.1)	23 (5.9)	
Night sweating	Yes	59 (95.2)	03 (4.8)	0.847
	No	356 (85.2)	24 (5.74)	
Comorbidity/Co-infection				
HIV infection	Positive	54 (94.7)	03 (5.3)	0.448
	Negative	154 (91.7)	14 (8.3)	
Diabetes mellitus	Yes	13 (92.9)	01 (7.1)	0.92
	No	376 (93.5)	26 (6.5)	
Hypertension	Yes	19 (100)	0(0.00)	0.24
	No	370 (93.2)	27 (6.8)	
Asthma	Yes	18 (100)	0(0.00)	0.253
	No	371 (93.2)	27 (6.8)	
Heart Disease	Yes	05 (100)	0(0.00)	0.553
	No	384 (93.4)	27 (6.6)	
Liver Disease	Yes	03 (100)	0(0.00)	0.647
	No	386 (93.5)	27 (6.5)	
Renal failure	Yes	01(100)	0(0.00)	0.792
	No	388 (93.5)	27 (6.5)	
Hormonal Disease	Yes	02 (100)	0(0.00)	0.709
	No	387 (93.5)	27 (6.5)	
Malignancy/Cancer	Yes	02 (100)	0(0.00)	0.709
	No	387 (93.5)	27 (6.5)	
Other risk factors				
Miner	Yes	04 (100)	0(0.00)	0.596
	No	383 (93.4)	27 (6.6)	
Ex-miner	Yes	07 (100)	0(0.00)	0.481
	No	380 (93.4)	27(6.6)	
Current smoker	Yes	10 (83.3)	02 (16.7	0.149
	No	377 (93.8)	25 (6.2)	
Ex-smoker	Yes	10 (90.9)	01 (9.1)	0.727
	No	377 (93.5)	26 (6.5)	
Prisoner/Prison staff	Yes	02 (100)	0(0.00)	0.708
	No	385(93.4)	27 (6.6)	
Migrant	Yes	8 (72.7)	03 (27.3)	0.005
	No	379 (94.0)	24 (6.0)	
Contact with TB patient	Yes	25 (92.6)	02 (7.4)	0.847
	No	362 (93.5)	25 (6.5)	
MDR-TB contact	Yes	07 (87.5)	01 (12.5)	0.489
	No	380 (93.6)	26 (6.4)	
Alcohol consumption	Yes, currently	59 (86.8)	09 (13.3)	0.042
	Yes, previously	52 (96.3)	02 (3.7)	
	Not at all	279 (94.6)	16 (5.4)	
Smokers	Yes, currently	11 (84.6)	02 (15.4)	0.174
	Yes, previously	11 (84.6)	02 (15.4)	
	Not at all	361 (94.0)	23 (6.0)	
Chewing Khat	Yes, currently	22 (88.0)	3 (12.0)	0.038
	Yes, previously	27 (84.4)	05 (15.6)	
	Not at all	341 (94.7)	19 (5.3)	

This was done based on both GeneXpert and culture results

**Table 6:** Showing adjusted odds ratio of those socio demographic factors, clinical presentation and risk factors having association in the univariate analysis, August 01, 2017- January 05, 2018

Variables	Results^a^		Adjusted Odds Ratio	*P-value*
	Positive Number (%)	Negative Number (%)	AOR (95% C.I)	
Occupation				
Farmer	01 (3.2)	30 (96.8)	1	
Daily Laborer	06 (7.9)	70 (92.1)	2.407 (0.255–22.723)	0.443
House wife	01 (1.1)	86 (98.9)	0.635 (0.033–12.243)	0.763
Driver	0 (0.00)	10 (100)	0 (0.00)	
Teacher	0 (0.00)	07 (100)	0 (0.00)	
Student	0 (0.00)	46 (100)	0 (0.00)	
Merchant	0 (0.00)	14 (100)	0 (0.00)	
Health Care Worker	01 (33.3)	2 (66.7)	68.7 (2.447–1929.15)	0.013
Government employee	02 (5.9)	32 (94.1)	2.38 (0.188–30.159)	0.503
Self-employed	07 (11.7)	53 (88.3)	3.031 (0.318–28.886)	0.335
Other	07 (16.3)	36 (83.7)	6.311 (0.662–60.122)	0.109
Sex				
Male	19 (10.2)	167 (89.8)	1	
Female	8 (3.5)	223 (96.5)	0.321 (0.097–1.062)	0.063
Loss of Appetite				
Yes	21 (8.6)	222 (91.4)	1	
No	6 (3.4)	168 (96.6)	0.778 (0.248–2.438)	0.666
Weight Loss				
Yes	20 (12.4)	141 (87.6)	1	
No	07 (2.7)	248 (97.3)	0.340 (0.108–1.069)	0.065
Shortness of Breath				
Yes	14 (10.4)	120 (89.6)	1	
No	13 (4.6)	270 (95.4)	0.493 (0.181–0.1.343)	0.167
Migrant				
Yes	03 (27.3)	08 (72.7)	1	
No	24 (06)	379 (94)	0.121 (0.20–0.730)	0.021
Alcohol Consumption				
Yes, currently	09 (13.2)	59 (86.8)	1	
Yes, previously	02 (13.7)	52 (96.3)	0.254 (0.046–1.399)	0.116
Not at all	16 (5.4)	279 (94.6)	0.956 (0.311–2.939)	0.937
Chewing Khat				
Yes, currently	03 (12)	22 (88)	1	
Yes, previously	05 (15.6)	27 (84.4)	1.324 (0.221–7.922)	0.759
Not at all	19 (5.3)	341 (94.7)	0.570 (0.127–2.565)	0.464

Key; ^a^ **=** the sum of GeneXpert and culture results

### Prevalence of smear negative multidrug resistant tuberculosis

A total of 26 smear negative, culture confirmed TB was detected. All clinical isolates were tested by using Genotype MTBDR plus Ver.2, first- line MGIT DST and Genotype MTBDRsl Ver.2. Among these isolates, 03 (11.54%) were MDR-TB (resistant to both isoniazid and rifampicin) and 07 (26.9%) were resistant to isoniazid. All rifampicin resistant (RR) TB by GeneXpert were found to be MDR-TB by Genotype MTBDR plus Ver. 2 and MGIT DST methods. Twenty three SNPT were isolated from new presumptive TB patients and two (8.7%) of them were rifampicin resistant. The remaining three SNPT were detected from previously treated patients and one (33.3%) of the isolates was rifampicin resistant. From 23 new SNPT isolates, 06 (26.08%) were resistant for isoniazid. From 03 (11.54%) isolates diagnosed from retreatment presumptive TB patients, 01 (33.33%) was resistant for isoniazid (*p* = 0.79). All of SNMDR TB 03/26 were detected from men (*p* = 0.264) and in the age group of 15–34 years old (*p* = 0.814). Drug susceptibility testing was performed for randomly selected 23 (88.46%) of the isolates for second line anti-TB drugs using Genotype MTBDRsl Ver.2, to check for any resistance but all the isolates were susceptible (Table 7).

**Table 7:** Genotypic and phenotypic drug susceptibility testing pattern of SNPT isolates, August 01, 2017- January 05, 2018

Drug susceptibility testing	Drugs		Status (Resistant/Sensitive)	
			Resistant No. (%)	Sensitive No. (%)
Genotype MTBDRplus Ver. 2	Rif		03 (11.54)	23 (88.46)
	INH		07 (26.9)	19 (73.1)
	MDR TB		03 (11.54)	23 (88.46)
	Drugs	Final drug concentration tested	Drug status (Sensitive/ Resistant)	
			Resistant No. (%)	Sensitive No. (%)
First line MGIT DST	STR	1.0 μg/ml	03 (15%)	17 (85%)
	INH	0.1 μg/ml	03 (15%)	17 (85)
	RIF	1.0 μg/ml	02 (10%)	18 (90%)
	ETM	5.0 μg/ml	01 (5%)	19 (95%)
	MDR TB		02 (10%)	18 (90)

First Line MGIT DST; the reason for variation with LPA is that 06 isolates from which 1 MDR Isolate, 03 were INH resistant by LPA was not finalized

Operationalized definitions

1. Smear Negative Pulmonary Tuberculosis (SNPT):- Is PTB that was initially negative for two (spot-spot) consecutive sputum smear examinations and later diagnosed positive by other advanced TB diagnostic tools [11]

2. Smear Negative Presumptive Pulmonary Tuberculosis (SNPPT) patients: - Are patients who have signs and symptoms and are presumptive to be diagnosed with pulmonary tuberculosis but two of their consecutive sputum samples are microscopically negative [37]

3. Alcohol consumption: is when a person takes alcohol every day and who was taking alcohol previously, but he/ she stopped drinking now

4. Smokers: are persons who smokes cigarettes every day and who was smoking previously, but he/ she stopped now

5. Contact with TB patient: according to WHO, these are people who have close contact with infectious TB and have high risk for infection with TB

## Discussion

According to WHO, more than 9 million people who become ill with TB each year; more than 3 million are not diagnosed, treated, or officially registered by national TB programmes. The emergence of MDR TB makes the situation worse. Collectively, these “missed” million TB patients are a global public health failure [7]. Similarly, diagnosis of MTB in developing countries largely depends on smear microscopy which has poor sensitivity [31], resulting in increased smear negative pulmonary tuberculosis (SNPT) that remains undiagnosed, or delay in diagnosis and treatment [10]. SNPT and SNMDR TB prevalence in Addis Ababa was not well known. Therefore, determining the magnitude and distribution of SNPT and smear negative MDR TB in different settings can contribute to the national efforts for fighting TB.

Our study findings showed that the prevalence of smear negative pulmonary tuberculosis among smear negative presumptive pulmonary tuberculosis patients was 27/418 (6.5%) (95% C.I, 4.103–8.816), which was Xpert MTB/ RIF confirmed and 26/408 (6.4%) %)(95% C.I, 4.003–8.743), by the collaborative diagnostic performance of BACTEC MGIT 960 and LJ culture. The difference between GeneXpert and culture results might be due to cross contamination during Xpert MTB/RIF sample processing or Xpert MTB/ RIF assay might detect dead remnant DNA from retreatment patients or harsh decontamination during culture processing. From the total, majority (55%) of the study participants were female, but more than two times SNPT (10.2%) were detected from men (*p* = 0.005) which is in line with the fact that TB is more common in men than women [1]. The present study revealed higher smear negative PTB prevalence than a report from Vietnam which was 5.6% (smear negative culture positive PTB) [32]. Our study reveals lower prevalence, as compared to a finding by a study conducted in 8 prisons in Amhara region, Ethiopia in the year 2013 that the overall prevalence of culture and/or Xpert test positive smear-negative pulmonary TB in the study was 8% [33]. Another study in Karamara hospital, Jigjiga, Ethiopia, conducted from December 2013 to May 2014, shown 7.9% SNPT prevalence; this result was the combined result of Xpert MTB/ RIF and culture-positive [34]. A study conducted in Adama, Ethiopia in 2013, also showed a higher SNPT prevalence that was 12.1% [16] than our study results and this study is similar with our study finding in that men were more affected by SNPT than females. Other study conducted in St. Peter TB specialized hospital Addis Ababa, Ethiopia in the year between November 2004 and October 2005, confirmed a higher prevalence that is 12.46% pulmonary TB isolated from smear negative pulmonary TB patients [18] than the present study. These differences may be due to different reasons like geographical variation, change in sample collection strategy, difference in time that the study conducted, change in TB diagnostic and treatment algorithm and diagnostic method differences. Another justification for these differences might be, our study tried to cover a wide range of facilities including private sites.

Although the study included all age groups, above two years, all (100%) of the SNPT were detected in the age group 15–64 years’ participants which are the economically productive age group of the population. This finding is in line with WHO estimation that states TB affects mostly adults in the economically productive age groups; around two-thirds of are estimated to occur among people aged 15–59 years [1].

According to the findings from the 26 isolates from smear negative presumptive pulmonary TB patients 03 (11.54%) were MDR TB. From these SNMDR TB isolates 02/23 (8.7%) were among new smear negative presumptive TB patients and 01/03 (33.3%) was from retreatment presumptive SNPT patients. Our study showed higher SNMDR TB prevalence in comparison with a study in Iran among tuberculosis patients in between the year 2011–2013, which was 4.3% (2.38% in the new TB treatment group and 23.1% in the retreatment groups) [35]. The present study demonstrated low MDR TB prevalence as compared to a finding by a study in Belarus which was 47% SNMDR-TB: 33% among new and 73% among previously treated patients [15]. But, our study showed high prevalence of SNMDR TB in comparison with a study in India that was conducted among newly diagnosed sputum-positive pulmonary tuberculosis between February 2008 and December 2009 demonstrated very low prevalence that was 1.13% MDR-TB [36]. The present study shows a very high SNMDR-TB prevalence even compared to the national MDR TB prevalence in Ethiopia which was performed among smear positive patients [6]. This is the first report in Addis Ababa. For instance, there was no smear negative MDR TB detected in a study conducted in St. Peter hospital, Addis Ababa, Ethiopia [18]. In our study among 27 Xpert MTB/ RIF confirmed *Mycobacterium tuberculosis* complex (MTBC) strains, 03 (11.11%) were Rifampicin resistant (RR) which is a bit higher compared to a study conducted in Karamara hospital, Jigjiga, Ethiopia which was 3/38 (7.9%); RR strains were identified from smear-negative culture-positive samples [32]. These variations of prevalence of SNMDR TB, and RR TB from our study result may be due to geographical location, population difference, time, study settings and method variation. Using Genotype MTBDRplus ver.2, Isoniazid (INH) mono resistance was 07/26 (26.9%). All RR SNPT isolates that Xpert MTB/ RIF assay diagnosed as RR were found to be MDR TB from which 01 (33.3%) MDR TB patients had TB contact history. Drug susceptibility testing was performed for 23 (88.46%) of the isolates for second line anti-TB drugs using Genotype MTBDRsl ver.2, to check for any resistance, but all isolates were susceptible. This study is different from other study conducted in Kenya, which showed resistance to first line and second line anti-TB drugs, but no MDR or XDR TB was detected [38].

Clinical features are significant in supporting the diagnosis of smear-negative TB even though it has poor sensitivity and specificity [31]. The present study to investigated clinical presentations, such as cough, fever, chest pain, loss of appetite, weight loss, shortness of breath, joint pain, abdominal pain, hemoptysis, and night sweating. But, none of this showed significant association with SNPT patients. Furthermore, cough, fever, weight loss, chest pain and night sweating are also symptoms in smear-positive patients, as it was reported in previous studies [33]. Shortness of breath, (AOR 0.493; 95% C.I, 0.181–0.1.343, *p* = 0.167), weight loss (AOR 0.340; 95% C.I, 0.108–1.069, *p* = 0.065) and loss of appetite (AOR 0.778; 95% C.I, 0.248–2.438, *p* = 0.666) are significant clinical features of SNPT patients in our study in the univariate analysis, but doesn’t have association in the multivariate model. However, another study reported that smear-negative patients were less probable to suffer from fever and weight loss than their smear-positive counterparts [39].

HIV comorbidity is a known confounder factor for the diagnosis of TB [8]. Our finding demonstrated 27% HIV positivity rate and 5.3% SNPT- HIV co-infection; but it doesn’t show a significant association and the comorbidity is much lower than the finding reported in Brazil which was 41% [9]. Our finding also showed that 7.1% SNPT among known diabetes mellitus patients and this finding is a bit higher compared to a study finding from St. Peter Hospital Addis Ababa, Ethiopia which was 6.7% [40]. This difference may be due to difference in study participants (they have done it on active TB patients), geographical coverage and time. From all demographic, behavioral and other risk factors that the study has assessed, sex (*p* = 005), work status or occupation (*p* = 0.006), being a migrant (*p* = 0.005), alcohol consumption (*p* = 0.042), chewing Khat (*p* = 0.038), has association with SNPT in a univariable analysis. A study conducted in Ethiopian prisons has showed alcohol consumption as a risk factor but chewing Khat was not associated with active TB disease [41]. Migrants have 0.121 times more likely to have smear negative pulmonary TB in comparison with non-migrants, (AOR 0.121; 95% C.I, 0.20–0.730, *p* = 0.021). The above factors needs to be further studied for the possible reasons of the association.

One limitation of the study is that chronic disease comorbidities are self-responded by the patient, not confirmed by diagnostic methods. The other one is phenotypic MGIT DST for Pyrazinamide (PZA) and for other 6 isolates for other first line drugs was not performed due to shortage of drugs and PZA sets.

## Conclusion

In this study a significant proportion of smear negative pulmonary TB was diagnosed. Furthermore, a high smear negative multi drug resistant (MDR) TB and other mono drug resistant TB prevalence was confirmed. Due to the limitations of smear microscopy which is used as a primary diagnostic tool, these TB strains are missed to be diagnosed and transmission continues in the community. Therefore, considering more sensitive diagnostic tools and drug susceptibility testing facilities in the peripheral TB diagnostic laboratories is crucial. Further studies on comorbidities and risk factors of SNPT are advised to be conducted in large scale.

## Data Availability

All original raw data is available with the corresponding author.

## Abbreviations

AFB: Acid fast bacilli
AIDS: Acquired immunodeficiency syndrome
BSC: Biosafety cabinet
DOTS: Directly observed treatment short course
DRS: Drug resistance surveillance or survey
DST: Drug susceptibility testing
EPHI: Ethiopian public health institute
EQA: External quality assurance
ETM: Ethambutol
GLI: Global laboratory initiative
HIV: Human immunodeficiency virus
INH: Isoniazid
LJ: Lowenstein jensen
LPA: Line probe assay
MDR: Multidrug resistant
MTB: *Mycobacterium tuberculosis*
NALC: N-Acetyl L-Cysteine
NaOH: Sodium hydroxide
NPV: Negative predictive value
NTM: Non tuberculosis mycobacteria
NTRL: National tuberculosis reference laboratory
PPV: Positive predictive value
PT: Proficiency testing
PTB: Pulmonary tuberculosis
RIF: Rifampicin
RR: Rifampicin resistant
SNMDR TB: Smear negative pulmonary multidrug resistance tuberculosis
SNPP: Smear negative pulmonary patients
SNPPTP: Smear negative presumptive pulmonary tuberculosis patients
SNPT: Smear negative pulmonary tuberculosis
SPPT: Smear positive pulmonary tuberculosis
STR: Streptomycin
WHO: World Health Organization
XDR: Extensively drug resistant
ZN: Ziehl Neelsen
